# Introduction: Marginalisation and the Medieval Church

**DOI:** 10.1080/03044181.2026.2642013

**Published:** 2026-03-13

**Authors:** Emmie Rose Price-Goodfellow, Tess Wingard

**Affiliations:** a Centre for Medieval Studies, University of York, York, UK; b Department of History, Trinity College, Dublin, Ireland

**Keywords:** Marginality, persecution, ecclesiastical history, gender, sexuality, disability, poverty, race

## Abstract

This article introduces the rationale for the special issue ‘The Medieval Church: From Margins to Centre’. It begins with a brief overview of the prior scholarship on medieval marginality which has informed the scope of this issue and its individual contributions. It then engages in a critical appraisal of the key concepts governing this issue, namely ‘the medieval church’ and ‘medieval marginalities’. Lastly, it outlines the contributions of each of the issue's articles, as well as the issue as a complete work, to the broader conversations around medieval marginalities.

What role did Christianity play in shaping the lives and experiences of marginalised groups in medieval societies? How did ecclesiastical bodies translate theories of difference into practice via the exercise of institutional power? In what ways could marginalised groups exert agency in their relations with the church? Turning to the matter of historiography, how have historians of medieval religion engaged with the intellectual provocations opened up by vibrant developments in theoretically informed fields such as premodern queer studies, trans and intersex histories, critical race studies, and crip theory? Likewise, how can the study of premodern marginalities be enriched by the major shifts in our understanding of the relationships between ecclesiastical institutions and the laity advanced by historians of the medieval church in the last thirty years? These are questions which have animated our thinking as medievalists, as collaborators, and as editors of this issue. They constitute an intellectual passion which we have shared for more than half a decade; we have made it our goal to build a scholarly culture which nurtures research into these areas through projects such as the *Ideology, Society, and Medieval Religion* online lecture series (active since January 2021) and *The Medieval Church: From Margins to Centre* conference, held at the University of York in June 2023; and of course, this present issue.^1^

The articles that follow explore the roles of the medieval church both in constructing marginality on a theoretical level, and in shaping the experiences of marginal groups. Our introduction aims to establish the intellectual groundwork for all that is to come. We begin with a brief overview of the prior scholarship on medieval marginality that has informed the scope of this issue and its individual contributions. We then engage in a critical appraisal of the key concepts of our investigation: what do we mean when we say ‘the medieval church’, or ‘medieval marginalities’? Lastly, we outline the contributions of each of our articles to the broader conversations around medieval marginalities, and the contributions of the issue as a whole.

The historiography of medieval marginality, the bulk of which is in English, is colossal, far beyond the scope of a brief introduction.^2^ Since the 1980s, studies on individual axes of marginality – relating to both the construction of categories of marginality and the lived experience of marginalised communities in medieval Latin Europe – have proliferated, including analyses of race and ethnicity,^3^ religious difference,^4^ disability and illness,^5^ poverty,^6^ gender and sexuality,^7^ and most recently, trans and intersex identity.^8^ To survey each of these fields of scholarship in turn would necessitate multiple essays; and in any case, the endeavour would be redundant, since each of the contributors to this issue provides a significantly richer invitation into their respective historiographies than we could offer here. In lieu of a comprehensive literature review, this introduction focuses on four medievalists – Bronisław Geremek, R. I. Moore, David Nirenberg, and Clare Monagle – who have each, in wildly divergent ways, proposed a kind of ‘unifying theory’ of medieval marginality that cuts across multiple categories, and whose work has in turn proved influential within scholarship. In their comprehensiveness, these works provide the context from which our definition of marginality and the rationale for this volume have emerged.

Originally published in Polish, Bronisław Geremek’s *The Margins of Society in Late Medieval Paris* (*Ludzie marginesu w średniowiecznym Paryżu, XIV-XV wiek*) came to the attention of the wider medieval community following its translation into French (1976) and then English (1987).^9^ Relying on legal records, but bringing in other sources including chronicles and hospital accounts, Geremek explores the lives of those who came before the secular courts in Paris from the thirteenth to fifteenth centuries.^10^ *The Margins of Society* offers no definition of marginality per se, beyond identifying the groups Geremek saw as existing on the fringes of society, primarily the very poor, criminals, sex workers, and the sick, particularly lepers.^11^ Although he did not explicitly define marginality in this context, all the groups Geremek focused on were generally marginalised on economic grounds – those who were very poor and did not work, the sick or disabled who could not work, and those such as criminals and sex workers who made a living through extra-legal means and so did not participate in the normal productive economy. This economic precarity led to legal marginalisation, as seen in the way vagrancy became criminalised in the fifteenth century. There was also a social element to this exclusion: family and neighbourhood bonds, already weaker in an urban environment, were further frayed by the Black Death, and in Geremek’s thinking, marginality was created through society’s inability to absorb fringe individuals or groups into these weak community networks. In this way, marginality is seen as a somewhat inevitable consequence of the socioeconomic conditions of late medieval Paris.

Geremek’s study pointed to several axes of marginalisation in medieval western Europe conditioned by economic status, and was followed by several important studies on poverty and charity that further explored this, including Michel Mollat’s *The Poor in the Middle Ages* (*Les pauvres au moyen âge*, 1978; English translation 1986).^12^ The notion that groups or individuals became marginal in medieval Europe when they could not be absorbed into the broader fabric of society or existing social networks was likewise taken up by Ephraim H. Mizruchi in his *Regulating Society* (1983), which deploys two medieval case studies, western monasticism and the rise of the beguine movement.^13^ In his structural account, surplus populations can be absorbed into society either through the expansion of existing groups (if there is openness to such expansion) or through the creation of new groups to deal with this surplus. This structural approach continued with perhaps the best-known book on medieval marginalisation, R. I. Moore’s *The Formation of a Persecuting Society* (1987).^14^ Here, Moore argues that the persecutions of a variety of groups in the twelfth and thirteenth centuries – heretics, Jews, lepers, sodomites – were linked. He sees marginalisation and persecution not as an inevitability in medieval society, but emerging due to specific developments in this period. In Moore’s account, the twelfth century saw the rise of a class of courtly clerical elites who constructed and persecuted these groups as social pollutants that had to be eradicated in order to extend the power of their own royal masters and to consolidate their own position. Thus various ‘threats’ to the social order were rhetorically conflated, and mechanisms of persecution developed. Despite what some of his critics have argued in the years since, Moore does not claim that the church was the sole or even principal driver of this ‘persecuting society’. Instead, he is more concerned with the growth of secular power, whilst acknowledging that the centralisation and consolidation of papal power meant that the church had incentives to persecute similar to those of secular princes.^15^

Moore’s work has been subject to a great amount of criticism, and some of his specific examples no longer stand – for example, much of his argument about the treatment of lepers, something he acknowledges himself in the second edition of *Formation of a Persecuting Society*.^16^ It has also been pointed out that the specifics of his arguments do not necessarily apply outside of the Anglo-Norman world he was studying.^17^ But as a book that does not take a persecutory mindset as a given in the Middle Ages, and so opens up the question of how and why the persecution of various groups arose, it remains useful. As John Arnold argues in his evaluation of Moore, what *Formation of a Persecuting Society* offers is a way of thinking about how ideologies moved between ecclesiastical and secular contexts in Latin Europe, and how the discourses and practices of persecution functioned in broader society and culture, and not only for the marginalised themselves.^18^ Moore’s work points to the need to study the processes and mechanisms of marginalisation and their origins; this turn away from the victims of persecution (the focus of Geremek’s study) to those who constructed discourses of marginalisation and put in place practices to persecute the marginalised has remained influential.

David Nirenberg’s *Communities of Violence* (1996), published just under a decade after *Formation of a Persecuting Society*, offers a highly critical response to Moore’s structural account of the development of marginalisation in the High Middle Ages. Where Moore had taken a pan-European approach, Nirenberg focuses on power relations between hegemonic Christian and minority Jewish and Muslim communities in the fourteenth-century Crown of Aragon. Nirenberg characterises Moore’s ‘persecuting society’ model as firstly, teleological: that is, his historical narrative of continually intensifying violence inevitably culminating in the industrialised genocides of the twentieth century misrepresents the ebbs and flows of medieval persecution and imposes a chronological unity that the historical evidence does not support.^19^ Secondly, he holds that it is reductionist: that is, Moore’s structural account of marginalisation privileges regions in which non-Christians were numerically few or even close to non-existent, and elides regions such as Iberia, where the presence of substantial populations of Jews and Muslims shaped a different set of political realities around interreligious and inter-ethnic relations than in, say, England, Scandinavia, or the Holy Roman Empire.^20^ Nirenberg argues instead that the picture that emerges in late medieval Aragon is not one of intensifying persecution driven by centralising institutions, but rather a status quo of ‘stabilising’ or ‘structural’ violence enacted by hegemonic Christians against marginalised communities in which both the crown and the nobility shared an interest in maintaining the protected status of Jewish and Muslim communities under their jurisdiction (while nonetheless marking them out as legally and politically subordinate).^21^ He regards the outbreaks of ‘cataclysmic’ violence against Jews and Muslims, such as the massacres of Jews in 1320–21, as aberrant events set apart from the status quo of systemic violence and not indicative of a broader tendency towards intensification. Nirenberg thus sees marginality as the product of a more constrained, structural form of violence, which enabled (uneasy) coexistence rather than overt repression.

Nirenberg’s analysis of the construction of marginality in late medieval Aragon provides several crucial insights which may be applicable in other historical contexts. In the first instance, his model of stabilising violence provides an alternative to the binary of persecution/tolerance. Nirenberg argues that institutional power over marginalised groups, as well as the informal power relations between hegemonic and marginal groups, were grounded in everyday acts of judicial and illicit violence which were nonetheless carefully circumscribed; an equilibrium of marginality, rather than a spiral of persecution. Within this analytical framework, there is perhaps greater scope to examine the agency of marginalised communities in negotiating with hegemonic institutions and groups and with navigating systems of oppression. Likewise, Nirenberg’s critique of teleology and reductionism in *longue durée* narratives such as that of Moore reinforces the importance of regional studies, and of nuancing broad-ranging structural accounts with consideration of the particular circumstances of local conditions – a balancing act which we hope to have achieved in bringing together such a range of topics here.

Where Geremek, Moore and Nirenberg frame their analyses of the construction of marginality in terms of the application of judicial and informal forms of violence, Clare Monagle centres the process of knowledge production in *The Scholastic Project* (2017). Though she focuses especially on the movement at its greatest period of intellectual output and vibrancy in the thirteenth century, Monagle examines the broader development of scholasticism as an academic movement across Latin Europe, circa 1100–1450, which emphasised dialectical reasoning, the incorporation of the works of Aristotle and his medieval Jewish and Islamic commentators, and the reconciliation of Aristotelian philosophy with Christian theology. She characterises scholasticism as not just a philosophical method but also a project of ideology akin to that of the Enlightenment.^22^ For Monagle, scholasticism entailed the creation of a ‘universal subject’ (male, Christian, clerical) whose experiences became embedded in the broader scholastic paradigm and its implicit assumptions about human nature and human societies.^23^ This universal subject was constructed through a process of exclusion, being defined in opposition to, for instance, women, heretics, and Jews; it entailed a hardening of categories, a more rigorous attempt to identify and classify forms of difference along gender, religious, and racial lines. Monagle shares with Moore a focus on clerics as the drivers of the construction of marginality, but where Moore’s thesis emphasised the role of secular clerics embedded within legal and governmental institutions as the key instigators of the persecuting society, Monagle’s structural analysis centres the work of knowledge production within the schools and universities.

Monagle’s thesis has value in its framing of scholasticism as more than simply a handmaiden of papal and secular power – as she notes, many scholastic intellectuals were regarded in their own lifetimes as controversial, heterodox figures who came into conflict with popes and bishops – seeing it instead as an autonomous ideological project which could nevertheless serve these interests given the right circumstances.^24^ Likewise, Monagle’s structural analysis of the creation of the universal subject through the exclusion of outgroups provides a nuanced account of the processes by which ideologies of marginalisation operate through, in Foucauldian terms, dictating the terms of the discourse: that is, by shaping the way that medieval societies think and write about categories of centre and margin.^25^ *The Scholastic Project* thus offers a model of medieval marginality grounded in the exercise of power through knowledge production, rather than violence.

Although the historians presented here, as well as other scholars who have further developed these ideas in their own work, have at times disagreed over the construction of medieval marginality, the four models they offer are not necessarily mutually exclusive. They are grounded in significantly divergent source bases; each of them privileges a different period as a key moment of historical change in the development of marginality (for Geremek, the period after the Black Death in particular; for Moore, the twelfth century; for Nirenberg, the fourteenth century; and for Monagle, the thirteenth century); and each of them is primarily concerned with a different process. All four models have influenced the contributions to this issue in different ways; each of the articles presented here should be read in light of these broader historiographical conversations regarding the operation of marginality in medieval societies.

But what, exactly, do *we* mean by marginality in the medieval context? As the editors of *Rethinking Medieval Margins and Marginality* (2020), a vibrant and self-reflexive recent intervention to the field of medieval marginality, note, this is an area where historians can unwittingly allow modern assumptions about the nature of different groups and their relation to structures of power to distort their understandings of the medieval margin:

it is necessary to consider context and subject matter very carefully before labelling medieval inhabitants as marginal. The demarcation of center and margin is inextricably linked with the assumption and assertion of power. The ways in which medieval people and modern scholars label the margin and the center has the potential to privilege, and thus, empower some at the expense of the others. It is important, therefore, to apply the term carefully in order to describe, but not reify, medieval power structures.^26^

Consequently, we must carefully define the parameters of marginality, especially in relation to the church. As our survey of prior historiography indicates, medievalists have often treated marginality as a broad yet circular category of difference or, to borrow Monagle’s phrasing, of exclusion: the marginal is whatever is not the centre. More specifically, in analyses of medieval texts and documents, the scholarly category of ‘marginal’ usually denotes any group whom the medieval source’s author regards, either implicitly or explicitly, as distinct from their own; this group is always conceived of within the source as being of a lesser status, whether socially, economically, or politically, from that of the author. Since the majority of surviving ecclesiastical sources from Latin Europe were composed by male Christians, under this definition the most straightforward marginal groups are thus women and non-Christians (Jews, Muslims, pagans, and heterodox Christians/‘heretics’) by virtue of their general exclusion from the process of textual production (though women could and at times did participate in this process).

Questions of class and social status are more complex: while medieval authors made much of literacy (particularly Latinity) as a marker of distinction, and often equated illiteracy with low social status, in practice, the varying capacities to compose written documents did not always map neatly onto the social hierarchy of medieval societies. Literate clerics might be economically impoverished, especially relative to more prosperous yet non-literate artisans; and the personal ability to read and write could be rare among those with economic and political power (though ameliorated through the employment of others to read and compose texts on their behalf).^27^ Poverty and illiteracy thus sit uneasily as categories of marginality in this schema. The same holds for categories of sexuality and disability, as contributions to this volume by Emmie Rose Price-Goodfellow and Hannah Kirby Wood demonstrate. Can queerness or disability be considered categories of marginality in the same ways as, for instance, womanhood or membership in religious minorities, when the latter categories were often much more existential barriers to participation in clerical textual production?

In editing this issue, we have constructed a definition of marginality drawing heavily on Monagle’s notion of the medieval universal subject, composed of three key constituent elements, which both addresses these epistemological challenges and aids the analytical work of investigating the relationship between the medieval church and marginal groups. For our purposes, marginality denotes those groups which are, firstly, *alienated from hegemonic clerical or monastic intellectual production*; in simple terms, they are groups who *are written about*, rather than who *write about* their own lives in ecclesiastical texts and documents. Secondly, they are *alienated as a class;* while individual hegemonic authors may be women, disabled, queer, etc., as collective groups and communities they are constructed as outsiders to clerical discourse. Lastly and most importantly, they are *subject to the imposition of structural power by ecclesiastical institutions*. The nobility, for instance, may satisfy the first two criteria, being largely alienated from intellectual production outside their roles as patrons, and being constructed in opposition to clerical identity in many texts; yet they as a class, they are self-evidently not subject to the imposition of structural power in ways that Jews, women, the poor, and so on, were. For our purposes, we are not concerned with alienation from economic production as an essential marker of marginality, per Geremek’s analysis, nor does numerical inferiority constitute a meaningful indicator of marginality by itself (one early reviewer of the proposal for this issue expressed scepticism as to whether women or the poor could be considered ‘marginal’ given that either of these groups constituted at least half of the population of medieval Europe at any given time; we hope we have now made it clear why this is a red herring). All of the essays in this issue explore the discursive construction and lived experiences of groups who meet this tripartite definition of marginality. We emphasise that we do not regard our definition as the only epistemologically valid one; rather it is one which we find to be the most useful for examining the role of the medieval church in the construction of marginality, and/or the interactions of marginalised peoples with the institutions and representatives of the medieval church.

This issue focuses primarily on five axes of marginality: disability, poverty, gender, sexuality, and religious difference. We do not claim to be comprehensive; there are of course other pertinent axes of marginality outside of these five, and even within our selection, we provide only a snapshot of certain experiences of different marginalised groups and their relationship with the medieval church. In particular, we have given less attention to the axis of race and racialisation than to others (though the essay by John Jenkins and Louise Hampson engages with the question of to what extent medieval Christian society constructed the Jew as a racialised as well as a religious marginal group); this is in large part due to a desire to avoid replicating existing and ongoing work. The construction of race within medieval intellectual production is an ever-expanding field, with influential recent studies by Geraldine Heng and Cord Whitaker among others exploring how Latin Christian categories of whiteness and Blackness emerged through clerical writings and interactions with non-Christian communities.^28^ Rachel Schine has moreover shown how comparable (though significantly divergent) categories of Blackness developed in medieval Islamic thought.^29^ A recent article by Michelle Armstrong-Partida and several essays in the volume *Rethinking Medieval Margins and Marginality* explore the development of categories of racial marginality and their impact on the lived experience of marginalised groups across the late medieval Mediterranean world and in early ethnographic writings.^30^ Our issue shares many of the same aims and methodological approaches as this vibrant and growing body of scholarship, and the essays that follow come to similar conclusions as much of this work in relation to other axes of marginality. We therefore hold that the work of this issue is complementary to what is being done in the field of medieval race studies; we aim instead to bring something new to the conversation by focusing on aspects of marginality such as poverty and transness which have historically been overlooked in structural analyses of medieval marginality, or were once scholarly concerns but have receded somewhat in recent years and stand to be revivified through modern critical lenses and in comparison with other axes of marginality.

In preparing this introduction, we made a conscious decision to avoid describing our account of medieval marginality and those of our contributors as ‘intersectional’. In its original formulation by the legal scholar Kimberlé Crenshaw, the theory of intersectionality holds that a person who inhabits multiple axes of marginality will not experience these identities as discrete categories; rather, each axis of marginality which they experience will inform the others.^31^ In Crenshaw’s model, a Black woman’s experience of misogyny cannot be separated from her racialisation as a Black person; conversely, her racialisation as a Black person inherently intersects with her marginalisation as a woman. The entire point of intersectionality as an analytical framework is that it focuses on the *intersections* of marginality, yet it is often misappropriated by researchers outside the field of Critical Race Theory as essentially synonymous with inclusivity or breadth of scope. For example, in his *Byzantine Intersectionality* (2020), Roland Betancourt explicitly cites Crenshaw’s intersectional framework, yet in practice his book compartmentalises different axes of marginality (race, transness, queerness etc.), only engaging in a truly intersectional analysis in the final chapter on Byzantine interpretations of the narrative of the Ethiopian eunuch of Acts 8.^32^ Conversely, Kevin Mummey’s innovative study of slavery in fourteenth-century Mallorca based on notarial records provides an example of genuinely intersectional research.^33^ Mummey demonstrates how the intersections of race, gender, and class shaped the experience of slave-owning and enslaved women in substantially different ways from their male counterparts, concluding that through their class and racial status, Christian women were as much a part of the machinery of slavery as men. As Mummey’s work demonstrates, intersectional analysis can be a valuable tool for deepening our understanding of marginality in the Middle Ages, but this must be done with intentionality and by paying attention to the *intersections* of identity; it cannot simply be applied as a label to describe research that explores different axes of marginalisation without meaningfully engaging with how they intersect. Intersectionality must therefore be an active scholarly choice embedded into the research praxis of a project from its very beginnings. Although our issue aims to examine multiple axes of marginality – and individual contributions sometimes explore overlapping forms of marginalisation – we make no claims to be intersectional; to do so would be an all-too-common misuse of Crenshaw’s work.

If we do not claim to present an intersectional view of medieval marginality with respect to the church, what is the aim of this volume? Our watchword in selecting contributions has perhaps been ‘granularity’ rather than intersectionality: specific instances of where a marginal identity was constructed or persecuted, as opposed, for instance, to Moore’s broad focus on the linked persecutions of various ostracised groups, or Geremek’s work on the variety of out-groups in late medieval Paris. In this, our approach is perhaps closest to Nirenberg’s, but also draws from Julie Singer’s call to think with experiences, rather than models.^34^ As Arnold has argued, if we move away from the notion of a unified persecuting discourse in the twelfth century, ‘we might find that the ‘persecuting society’ was one in which there was a greater distribution of identities rather than simply insiders and all-those-lumped-together-as- outsiders.’^35^ The contributions in this volume each examine one axis of marginalisation in one specific instance – poor clerics in late medieval England, the trans experience in the Cistercian cloister, the lives of Jews in thirteenth-century York, the construction of queerness as an existential threat in the aftermath of the Black Death in England – and so study one marginalised identity group and their interactions with the organs of the church.

Now is perhaps a pertinent moment to address the question of why this issue focuses on the church in relation to marginality (a different approach to that taken by Geremek, Moore, and Nirenberg, though less so that of Monagle) and, crucially, what we mean by ‘the church’. We define the church in the medieval Latin west as the aggregate set of institutions under the ultimate authority of the Pope, involved in both ecclesiastical knowledge production and the development and implementation of ecclesiastical law. This definition brings together the two elements of knowledge and power which, *pace* Foucault, dictated the construction of marginality in theory and its implementation in practice.^36^ ‘The church’ can mean the Pope himself and his curia; it can be the networks of archbishops and bishops within any given province; it can be a diocesan court, an individual religious house, a secular church or cathedral, or a school or university. ‘The church’ was never a monolithic organisation, and different individuals and institutions within it held competing interests and came into conflict over the course of the later Middle Ages (not least in the confrontations between the monastic and mendicant orders). Moore has frequently been criticised for overstating the unity of ‘persecuting’ discourses in the twelfth and thirteenth centuries, and we are alive to the importance of focusing on the particular and not eliding differences.^37^ Each of the articles in this issue draws out a targeted focus on specific institutions within the church and their relationships with marginal groups, while not losing sight of the bigger picture – that these individual institutions composed the collective body of the church. By the twelfth century, institutions staffed by clerics and monastics – that is, religious houses, schools, and universities – had become the primary site of intellectual production in Latin Europe. The church in this period possessed one of the most sophisticated document-based bureaucracies in Europe, which secular polities were only beginning to match in output and complexity. Both the papacy itself and individual bishoprics and religious houses were major landowners and agents within both international and regional politics across the continent, and power was becoming increasingly centralised and consolidated. The church maintained an increasingly complex trans-national system of canon law. High and late medieval Europe was a multipolar world, and the church was indisputably a site of significant institutional power – although one of several – and therefore unavoidable in our contributors’ writing histories of marginality.

The case study approach taken by the articles in this issue reveals not just how discourses of marginalisation were constructed by the medieval church, but how these discourses played out on the ground, and crucially how members of marginalised groups themselves responded to these discourses and mechanisms of persecution. Here, we are indebted to the Black feminist thought of bell hooks as articulated in her classic text *Feminist Theory: From Margin to Center* (1984), to which the subtitle of this special issue is gesturing.^38^ hooks’ work grants us a crucial insight into the mutually constitutive relationship between marginality and power: marginality is *complex* and not a simple binary of marginal/dominant. A subject can simultaneously be marginal and also operate within systems of power. They may use their position within these systems to negotiate their own marginal status, but this act of negotiation entails the disruption or dismantling of the systems of power and hierarchy through which marginality is constituted. To bring this discussion back around to the Middle Ages, per the ‘social church’ thesis, there are precedents for thinking about non-hegemonic – though not necessarily marginalised – voices in church documents.^39^ Rather than a marginalised identity being imposed from above by those in authority or dominant social groups (as is the case in the four studies explored at the start of this introduction), this approach demonstrates that these identities and their meanings could be constructed in a dialogue between the marginalised and the church.

For example, Edmund van der Molen’s article reveals how canonisation processes gave disabled people the opportunity to voice and construct their own narratives about their bodies; likewise, Emmie Rose Price-Goodfellow’s contribution explores how Cistercian understandings of gender and religious life could provide opportunities for trans individuals in the cloister to live authentically. It should go without saying that the medieval church and its representatives exercised greater power than members of marginalised groups (although, as we have already noted, ecclesiastical agents could themselves experience one or multiple axes of marginality), not least because of the monopoly on the production of knowledge, but this does not imply that marginalised individuals were always or necessarily passive objects or victims. In Moore’s formulation the marginalised themselves are affected by the workings of power; but as the contributions to this volume show, they were also active subjects who worked to improve their lives and narrate their own experiences, albeit in straitened circumstances.

This volume thus makes a methodological intervention in the field of medieval marginality studies, demonstrating ways to read marginality from church-produced sources. One of the great challenges when studying persecuted medieval groups will always be the dearth of sources produced by individuals from those groups, which has often led to a scholarly focus on the construction of marginality or the persecution of the marginalised (as per Moore, Nirenberg and Monagle). Each article in this volume brings a different theoretical perspective to bear on the selection and interpretation of sources, showing how these new lenses enable historians to uncover the agency of marginalised individuals even in non-marginal sources. This kind of reading ‘against the grain’ to uncover marginalised voices and experiences has long been a feature of scholarship on medieval women; this Special Issue presents modes of reading to access the voices of other, less-studied, marginalised groups, to whom this approach has not always been applied. ^40^

The contributions here combine three elements: a theoretical or critical lens applied to the selection and interpretation of sources, a rigorous understanding of the norms of the source type under consideration (including considerations of author, production context, and audience), and attention to the historical circumstances in which the sources were produced. This approach – similar to that employed by several of the essays in *Rethinking Medieval Marginality* – answers many of the criticisms of previous studies of marginalised individuals or groups in the Middle Ages, which often revolve around a lack of care paid to the specific historical circumstances of the moment under consideration.^41^ The diversity of case studies in this volume further demonstrate the applicability of this methodology to the study of a wide variety of axes of medieval marginality.

This volume is arranged broadly thematically. The articles by Edmund van der Molen and Anne Bailey both consider miracle narratives through the lens of disability studies, applying modern sociological theories to medieval texts to uncover the agency of the subjects of miracle stories. Rather than relying on modern models of disability, van der Molen takes up the call to reorient medieval disability studies towards the role of impaired individuals themselves in negotiating their position in society, and, in this case, their relationship with the church. Through the lens of Alison Kafer’s ‘crip futurity’, van der Molen reads canonisation depositions to uncover the futures that disabled individuals imagined for themselves, outside of the dominant church-imposed binary of cure or death.

Anne Bailey’s contribution studies pilgrimage and rehabilitation as one option that medieval society offered to the sick, disabled, very poor, or socially disadvantaged for their reintegration into the centre. Analysing English miracle collections from the long twelfth century, Bailey uses ritual theory to explore the wider sociological significance of the ‘healing journey’ of pilgrimage, as an act that healed dominant society as well as marginalised individuals. She further explores how these narratives could have both warned and comforted the marginalised, providing them with models of rehabilitation and integration into the wider Christian community.

Hannah Kirby Wood’s article likewise looks at disability and poverty, through the lens of institutional church support for poor stipendiary clerics in late medieval England. Using bishops’ registers, taxation records, and the charters of charitable institutions, she examines the vocational crisis faced by unbeneficed clerics, their response to it, and the support offered to them, asking to what extent the church attempted to alleviate or even acknowledge the problem. By studying poor clerics as marginalised, Kirby Wood destabilises the boundary between ‘margins’ and ‘centre’; she takes this further by examining ‘institutional’ support, such as minimum wages for vicars; support offered by church officials of their own accord; and measures enacted by clerics themselves, including the unbeneficed.

John Jenkins’s and Louise Hampson’s article takes on another axis of marginalisation in medieval England, exploring the participation of the Jewish community in the civic life of thirteenth-century York. Jenkins and Hampson argue that studies of the racial and religious othering of England’s Jewish community tend to overestimate the ability of authorities to exert control at a local level, while underestimating the fragmentary nature of authority in twelfth- and thirteenth-century English towns and cities, and fail to compare the Jewish experience to that of their fellow Christian citizens. By treating medieval Jewish urban life not just as Jewish but as British history, they argue persuasively for the important role played by the Jewish community of York after the massacre of 1190, and demonstrate sustained cooperation between Jews and their Christian neighbours. They argue that while it may have been rhetorically marginalised by church teaching, the Jewish community of York was central to the ordinary social life of the city.

Tess Wingard’s article focuses on the treatment of another marginalised group, queer people, in medieval England, exploring the church’s response to sodomy after the Black Death by using sermons and bishops’ registers as key sources. Drawing on historical and sociological perspectives on the cultural framing of pandemics as ‘moral crises’, Wingard argues that the medieval church saw moral reform as the foundation of their plague response, and in particular the containment of sexual sin, primarily sodomy. She demonstrates how existing theological notions were appropriated in this response and disseminated to the laity through sermons, providing a pathway along which ecclesiastical ideologies moved into secular contexts and were put into practice on the ground, and so may have shaped the actual lived experience of queer people in England in the aftermath of the plague.

The final article, by Emmie Rose Price-Goodfellow, explores how Cistercian notions of gender and religious life could encompass more than a strict, cisgender, male-female binary. Analysing an exemplum about a trans monk, Brother Joseph, from Caesarius of Heisterbach’s *Dialogus miraculorum*, she argues that Caesarius constructed his subject as a trans monk even if not precisely a trans man, and that this construction was central to the exemplarity of the Joseph’s story. Furthermore, she shows that these gender-fluid readings are not a modern imposition, but are only fully legible when situated within the broader context of Caesarius’s writings and Cistercian conceptions of gender and the religious life in this period.

As the historiographical survey in this introduction has demonstrated, the field of medieval marginality studies is flourishing, with more publications coming every year. This vibrancy perhaps shows how medievalists are being influenced by and responding to the world around us. Necessary and long-overdue fights for social justice are occurring around many modern axes of marginalisation: protests for the bodily autonomy of women and the transgender community; against racism and the police brutalisation of people of colour, especially Black people; against colonial violence; for the rights of migrants and refugees; for climate justice. Amidst a global lurch to the authoritarian right, these fights are more urgent than ever. As people in this world – especially if we are marginalised ourselves – it is inevitable that medievalists will be drawn to studying similar themes in the context of the Middle Ages. Now, the contributions to this journal issue were not written to speak to the contemporary moment, and our goal is not to provide answers for modern political movements. Nonetheless, the present and the past – even the medieval past – are always in dialogue. We hope that this volume provides intellectual and imaginative space for every scholar grappling with the issue of marginalisation, be it medieval or modern.

## Footnotes

^1^ Ideology, Society, and Medieval Religion: Impositions and Negotiations, eds. Emmie Rose Price-Goodfellow and Tess Wingard, https://medreligion.wordpress.com/ (accessed 2 March 2026).

^2^ We note here and in subsequent footnotes key contributions in other languages to the historiography of medieval marginality such as Guy-H. Allard, ed., *Aspects de la marginalité au moyen âge* (L’Aurore, 1975); Bernd-Ulrich Hergemöller, *Randgruppen der spätmittelalterlichen Gesellschaft* (Fahlbusch, 1994); Giacomo Todeschini, *Visibilmente crudeli: Malviventi, persone sospette e gente qualunque dal medioevo all'età moderna* (Il Mulino, 2007); Jean-Pierre Leguay, *Pauvres et marginaux au moyen âge* (Éditions Jean-Paule Gisserot, 2009).

^3^ Geraldine Heng, *The Invention of Race in the European Middle Ages* (Cambridge University Press, 2018); Cord J. Whitaker, *Black Metaphors: How Modern Racism Emerged from Medieval Race-Thinking* (University of Pennsylvania Press, 2019); Claire Weeda, *Ethnicity in Medieval Europe, 950–1250: Medicine, Power and Religion* (York Medieval Press, 2021).

^4^ John H. Arnold, *Inquisition and Power* (University of Pennsylvania Press, 2001); Robert Chazan, *Reassessing Jewish Life in the Medieval Europe* (Cambridge University Press, 2010).

^5^ Carole Rawcliffe, *Leprosy in Medieval England* (Boydell & Brewer, 2006); Irina Metzler, *Disability in Medieval Europe: Thinking about Impairment in the High Middle Ages, c.1100–c.1400* (Routledge, 2006); Irina Metzler, *Fools and Idiots? Intellectual Disability in the Middle Ages* (Manchester University Press, 2016)

^6^ Sharon Farmer, *Surviving Poverty in Medieval Europe*: *Gender, Ideology and the Daily Lives of the Poor* (Cornell University Press, 2001); Sharon Farmer, ed., *Approaches to Poverty in Medieval Europe: Complexities, Contradictions, Transformations, c.1100–1500* (Brepols, 2016).

^7^ Dyan Elliott, *Fallen Bodies: Pollution, Sexuality and Demonology in the Middle Ages* (University of Pennsylvania Press, 1998); Katherine J. Lewis, Noël James Menuge, and Kim M. Philips, eds., *Young Medieval Women* (Sutton Publishing, 1999); Ruth Karras, *Unmarriages: Women, Men, and Sexual Unions in Medieval Europe* (University of Pennsylvania Press, 2012); Tom Linkinen*, Same-Sex Sexuality in Later Medieval English Culture* (Amsterdam University Press, 2015); Martin Ingram, *Carnal Knowledge: Regulating Sex in England, 1470–1600* (Cambridge University Press, 2017).

^8^ Clovis Maillet, *Les genres fluides: De Jeanne d’Arc aux saintes trans* (Arkhe, 2020); Leah DeVun*, The Shape of Sex: Nonbinary Gender from Genesis to the Renaissance* (Columbia University Press, 2021); Greta LaFleur, Masha Raskolnikov, and Anna Klosowska, eds*., Trans Historical: Gender Plurality before the Modern* (Cornell University Press, 2021); Alicia Spencer-Hall and Blake Gutt, eds., *Trans and Genderqueer Subjects in Medieval Hagiography* (Amsterdam University Press, 2021).

^9^ Bronisław Geremek, *The Margins of Society in Late Medieval Paris*, trans. Jean Birrell (Cambridge University Press, 1987). Published in French as *Les marginaux parisiens aux XIVe et XVe siècles* (Flammarion, 1976), and originally in Polish as *Ludzie marginesu w średniowiecznym Paryżu, XIV-XV wiek* (Ossolineum, 1971).

^10^ Geremek’s study focuses on the Châtelet, the principal criminal court in Paris, supplemented with evidence from manorial courts; unlike many other historians of marginality, he did not make extensive use of material from the ecclesiastical jurisdiction.

^11^ Barbara A. Hanawalt, ‘Review*: The Margins of Society in Medieval Paris*’, *Speculum* 64, no. 4 (1989): 947–49 (947), for a critique of Geremek’s lack of definition.

^12^ Michel Mollat, *The Poor in the Middle Ages: An Essay in Social History*, trans. Arthur Goldhammer (Yale University Press, 1986).

^13^ Ephraim H. Mizruchi, *Regulating Society: Marginality and Social Control in Historical Perspective* (Free Press, 1983).

^14^ Robert I. Moore, *The Formation of a Persecuting Society: Power and Deviance in Western Europe, 950–1250* (Blackwell Publishers, 1987).

^15^ R. I. Moore, *Formation of a Persecuting Society*, 2nd ed., 146–66.

^16^ Moore, *Formation of a Persecuting Society*, 2nd ed., 180–83.

^17^ John H. Arnold, ‘Persecution and Power in Medieval Europe: The Formation of a Persecuting Society by R. I. Moore’, *The American Historical Review* 123, no. 1 (2018): 165–74 (172).

^18^ Arnold, ‘Persecution and Power’, 171.

^19^ David Nirenberg*, Communities of Violence: Persecution of Minorities in the Middle Ages* (Princeton University Press, 1996), 3–7.

^20^ Nirenberg, *Communities of Violence*, 241–45.

^21^ Nirenberg, *Communities of Violence,* 231–49.

^22^ Clare Monagle, *The Scholastic Project* (Arc Humanities Press, 2017), 8–11.

^23^ Monagle, *The Scholastic Project,* 11–13.

^24^ Monagle, *The Scholastic Project,* 43–50.

^25^ For the classic account of Foucault’s notion of discourse and its role in the relationship between power and knowledge production, see Michel Foucault, *The History of Sexuality*, trans. Robert Hurley, vol. 1 (Penguin, 1978), 17–73.

^26^ Ann E. Zimo, Tiffany D. Vann Sprecher, Kathryn Reyerson and Debra Blumenthal, eds., *Rethinking Medieval Margins and Marginality* (Routledge, 2020), 8.

^27^ The classic account of the rise and importance of literacy and its use by the elite in western Europe is of course M. T. Clanchy, *From Memory to Written Record, England, 1066–1307* (Blackwell 1979). On poor clerics, see Kathryn Kerby-Fulton, *The Clerical Proletariat and the Resurgence of Medieval English Poetry* (University of Pennsylvania Press, 2021).

^28^ Heng, *The Invention of Race*; Whitaker, *Black Metaphors.* Since literary studies has proved more receptive than other disciplines to engaging with explorations of race and racialisation in the European Middle Ages, the canonical, most-cited book-length studies on this topic have written by literary scholars (such as Heng and Whitaker). However, there have also been important historical studies on the topic, such as Peter Biller, ‘Black Women in Medieval Scientific Thought,’ *Micrologus* 13 (2005): 477-92, or the collected essays in Miriam Eliav-Feldon, Benjamin Isaac and Joseph Ziegler, eds., *The Origins of Racism in the West* (Cambridge University Press, 2009).

^29^ Rachel Schine, *Black Knights: Arabic Epic and the Making of Medieval Race* (University of Chicago Press, 2024).

^30^ Michelle Armstrong-Partida, ‘Race, Skin Colour, Enslavement and Sexuality in the Late Medieval Mediterranean,’ *Journal of Medieval History* 50, no. 4 (2024): 477–99; in Zimo et al., *Rethinking Medieval Margins,* Lori De Lucia, ‘The Space Between Borno and Palermo: Slavery and Its Boundaries in the Late Medieval Saharan-Mediterranean Region’; Sierra Lomuto, ‘Race and Vulnerability: Mongols in Thirteenth-Century Ethnographic Travel Writing’; Kevin Mummey, ‘Measuring the Margins: Women, Slavery, and the Notarial Process in Late Fourteenth-Century Mallorca’; and Ann E. Zimo, ‘How Marginal is Marginal? Muslims in the Latin East’.

^31^ Kimberlé Crenshaw, ‘Demarginalizing the Intersection of Race and Sex: A Black Feminist Critique of Antidiscrimination Doctrine, Feminist Theory and Antiracist Politics’, *University of Chicago Legal Forum* 1 (1989): 139–67.

^32^ Roland Betancourt, *Byzantine Intersectionality: Sexuality, Gender and Race in the Middle Ages* (Princeton University Press, 2020), 14-17 and 161-203. For a fuller critique of Betancourt’s use of intersectionality, see Stavroula Constantinou, review of R. Betancourt, *Byzantine Intersectionality* in *Peregrinations: Journal of Medieval Art and Architecture* 7 (2021): 363–74 (363–64).

^33^ Mummey, ‘Measuring the margins’, 111–28.

^34^ Julie Singer, ‘Disability and the Social Body’, *postmedieval* 3, no. 2 (2012): 135–41 (137).

^35^ Arnold, ‘Persecution and Power’, 173.

^36^ Our introductory discussion, as well as the essays which follow in this Special Issue, focus exclusively on western Christianity. The diversity of traditions and institutional structures in medieval eastern Christianity (including the Orthodox church, the Coptic church, the Chaldean church and others) means that we cannot assume that any analysis of the relationships between marginality and the Latin church necessarily also holds true for the eastern churches. For an introductory overview of eastern Christianity in the Middle Ages, see Michael Angold, ed., *The Cambridge History of Christianity*, vol. 5, *Eastern Christianity* (Cambridge University Press, 2006).

^37^ Arnold, ‘Persecution and Power’, 173.

^38^ bell hooks, *Feminist Theory: From Margin to Center* (South End Press, 1984).

^39^ See for example, John H. Arnold, *Belief and Unbelief in Medieval Europe* (Hodder Education, 2005); Ian Forrest, *Trustworthy Men: How Inequality and Faith made the Medieval Church* (Princeton University Press, 2018); Bronach Kane, *Popular Memory and Gender in Medieval England: Men, Women and Testimony in the Church Courts, c. 1200–1500* (Boydell & Brewer, 2019).

^40^ See for example Judith M. Bennett, *Women in the Medieval English Countryside: Gender and Household in Brigstock before the Plague* (Oxford University Press, 1987; P.J.P. Goldberg, *Women, Work, and Life Cycle in a Medieval Economy: Women in York and Yorkshire c.1300-1520* (Oxford University Press, 1992); Sara M. Butler, *The Language of Abuse: Marital Violence in Later Medieval England* (Brill, 2007)

^41^ See, for example, Nirenberg and others’ criticism of Moore, discussed earlier in this introduction. For criticism of this approach with regards to specific axes of marginalisation, see the consideration of Geraldine Heng’s work by Jenkins and Hampson in this volume, or van der Molen’s critique of the application of modern models of disability to medieval individuals.

